# Why do general practitioners prescribe antidepressants to their patients? A pilot study

**DOI:** 10.1186/1751-0759-8-17

**Published:** 2014-07-30

**Authors:** Alain Mercier, Isabelle Auger-Aubin, Jean-Pierre Lebeau, Matthieu Schuers, Pascal Boulet, Paul Van Royen, Lieve Peremans

**Affiliations:** 1 Department of General Practice, Rouen University and CIC Inserm 0204, University of Rouen, Rouen, France; 2 Department of General Practice, Denis Diderot Paris 7 University, Paris, France; 3 Department of General Practice, Tours University, Tours, France; 4 Department of Primary and Interdisciplinary Care, Faculty of Medicine and Health Science, University of Antwerp, Antwerp, Belgium; 5 Department of Public Health, Vrije Universiteit Brussel, Brussels, Belgium; 6 Department of Family practice, Rouen University, Faculty of Medicine, 20 Bd Gambetta, Rouen 76000, France

**Keywords:** Antidepressants, Primary care, General practitioner, Prescription, Off label use

## Abstract

**Background:**

The frequency of antidepressant (ADs) prescription is high, with general practitioners (GPs) responsible for about 80% of the prescriptions. Some studies considered prescriptions meet DSM criteria, while others stress inadequate use. The importance of biological and psychosocial determinants of GP prescription behaviour remains little explored. We aimed to describe the importance of these biological and psychosocial determinants and their weight in the daily practice of GPs’.

**Methods:**

During a week chosen at random, 28 GPs collected the AD prescriptions made within the previous six months, regardless of the reason for the patient contact. Bio psychosocial and AD treatment characteristics were recorded for all patients. In a random sample of 50 patients, patient characteristics were assessed via a structured face-to-face interview with the GP.

**Results:**

The frequency of AD prescription was 8.90% [3.94 -17.02]. The GPs initiated 65.6% [60.1-70.8] of the prescriptions. The rate of AD prescription for non-psychiatric conditions was 18%. Patients had from 1 to 9 conditions, showing a high level of multi-morbidity. There was a strong influence of past medical history and contextual problems, such as work related problems.

**Conclusion:**

AD prescription is related to complex contextual situations and multi-morbid patients. GPs use a bio psycho social approach, rather than a purely biological assessment. Awareness of these influences could improve prescription by GPs.

## Background

Antidepressant (AD) sales increased sevenfold in industrialized countries from 1980 to 2008 and have remained rather stable since then [1-3]. In the overall population, the 12-month rate of AD consumption ranges from 6% to nearly 10% [3-5]. The AD consumption is very high in France (9.7%) and in the USA (10.7%) [6-8]. In all countries, General Practitioners (GPs) are responsible for about 80% of these prescriptions [2,9]. This high level of consumption could be explained by a greater demand for medical care and an increased number of patients treated for depression [10,11]. The increasing rate of long-term treatment of depressed people could also explain this consumption [12]. ADs are also prescribed for other mental disorders, such as anxiety [13] and for minor depression [3,14]. ADs could also be adequately used in treating non-psychiatric conditions such as pain [15]. Some studies have shown that most patients (86-95%) diagnosed as depressed and treated by their GP with ADs met the DSM-IV depression criteria of a major depressive disorder (MDD) [16,17]. Others showed that the rate of off-label prescriptions varies between 20% and 60% of all prescribed ADs [18,19]. They are also prescribed in an inadequate manner, such as a too short duration of treatment [20] or in a sub-therapeutic dosage [3]. All these discrepancies suggest a lack of data, probably related to the complex real-life situations seen in primary care: Qualitative research has revealed that GPs usually prescribe ADs for non-psychiatric or mixed conditions, sometimes off-label, influenced by overall patient characteristics [21,22]. The relative influence of these factors on AD prescription among GPs remains unexplored, particularly regarding the characteristics of the patients’ condition and their social environment. The main aim of this pilot study is to more precisely describe the importance of these biological and psychosocial determinants as they relate to GPs in their daily practice.

## Methods

AD prescriptions and the characteristics of patients who had been prescribed an AD in the last six months were gathered.

### Pilot study participants and settings

The study was conducted in the Normandy region of France, in the northwestern area of the country. Like many regions, it includes industrialized cities (e.g. Rouen, Le Havre) as well as rural areas and has an overall population of 1.9 million inhabitants. Access to the health care system does not differ from the usual standard of the country: In France, GPs are the first medical contact for the patients and provide open access to its users, dealing with all health problems regardless of the age, sex, or any other characteristic of the person concerned. A three year specialized curriculum, (or official equivalency) is mandatory to practice as a GP. All GPs from our local research network were invited to participate in the study. Those invited all worked full-time at a primary ambulatory care practice, only seeing outpatients. None of the physicians worked in specialized or secondary care settings (e.g. hospital, clinics).

### Data collection

The GPs collected the data during a week chosen at random between December 2011 and March 2012. We included all patients who had been prescribed an AD within the previous six months, including renewals and new prescriptions, regardless of the reason for contact, and whether or not this prescription was continued later on. Patient characteristics (gender, age, socio-professional category and marital status) and the AD treatment characteristics (dosage, estimated starting date, and initiator of the prescription) were recorded. In order to focus in depth on all biological and psychosocial characteristics, we randomly selected from each GP two patients who had been prescribed an AD within the previous six months. The selection was stopped when a total of 50 patient files had been drawn. Once these files were selected, the patients’ characteristics were assessed via a structured face-to-face interview with the GP. The assessment included risk factors for depression and on-label and off-label conditions for AD prescription. All other characteristics encountered during our previous qualitative analysis of AD prescription by GPs’ were also collected [22]. Those characteristics were related to the psycho-social context of the patient (e.g. relational conflicts in the family, social loneliness, see Table 1), past history, and on-going conditions, as they were perceived by the GPs. During the interview, the GPs were asked to use information from the patient’s file and to rate the influence of each characteristic for each AD prescription (“0” for no influence on their AD prescription, “1” for a slight influence and “2” for a major influence). The aim was to weight the relative influence of the factors for an AD prescription according to the GP himself. We wanted to point out the influence of less prevalent factors than psychiatric disorders (e.g. life events, pain conditions).

**Table 1:** Diagnosis, psychosocial context, past history, and ongoing conditions (n = 50)

		**All patients n = 50**	**All patients with at least one psychiatric diagnosis**^**(1) **^**n = 41**	**All patients with a depressive episode**^**(2) **^**n = 31**	**All patients with a major depressive episode (intense)**^**(3) **^**n = 20**	**All patients with no psychiatric diagnosis n = 9**
**Gender**	Female	**34**	**29**	**21**	**13**	**5**
**Age**	Mean/Range	**52.8 [19–91]**	**50.6 [19–91]**	**48.8 [19–91]**	**45.8 [19–77]**	**62 [40–84]**
**Job**	Professional activity	**26**	**22**	**17**	**11**	**3**
**Socio- professional class**	Low income	**19**	**17**	**11**	**7**	**2**
	Intermediate to high income/	**15**	**12**	**11**	**9**	**3**
	retirement	**16**	**12**	**9**	**4**	**4**
**Marital status**	Living together/Married	**21**	**15**	**17**	**4**	**8**
	Single, Divorced, separation	**19**	**17**	**15**	**12**	**0**
	Widowed	**10**	**9**	**9**	**4**	**1**
	Relational conflicts in family	**18**	**17**	**15**	**11**	**1**
**Current context**	Disability (official status)	**8**	**6**	**5**	**3**	**2**
	Social loneliness/Socially isolated patient	**5**	**5**	**5**	**5**	**0**
	Work-related problems	**17**	**14**	**10**	**5**	**3**
	Legal problems/Current legal situation	**4**	**4**	**4**	**4**	**0**
	Recent bereavement/Recent death in the entourage	**12**	**9**	**9**	**5**	**3**
	Position of family care giver	**7**	**7**	**5**	**3**	**0**
	Patient physically or psychologically dependent	**7**	**7**	**6**	**4**	**0**
	Facing discrimination	**5**	**5**	**4**	**2**	**0**
	Current tobacco consumption	**14**	**12**	**10**	**6**	**2**
	Excessive alcohol consumption	**3**	**3**	**2**	**2**	**0**
	Other addictions (Opiates, gambling, etc.)	**6**	**5**	**4**	**3**	**1**
	Substitution therapy (methadone, buprenorphine)	**1**	**1**	**1**	**1**	**0**
**Number of contextual factors by patient Mean/Range**		**2.1 [0–6]**	**2.31 [0–6]**	**2.7 [0–6]**	**2.6 [0–6]**	**1.33 [0–4]**
**Past history of patient**	Past history of suicide attempt	**6**	**6**	**4**	**3**	**0**
	Tobacco consumption	**18**	**15**	**12**	**8**	**3**
	Past history of excessive alcohol consumption	**7**	**6**	**5**	**5**	**1**
	Other addictions (e.g. opiates, gambling)	**4**	**3**	**4**	**3**	**1**
	Past history of depression (different from current episode)	**19**	**18**	**14**	**8**	**1**
	Past history: Hospitalised in psychiatric service, whatever the reason	**5**	**5**	**3**	**2**	**0**
	Family with psychiatric conditions	**10**	**9**	**5**	**5**	**1**
	Past history of AD prescription	**23**	**22**	**15**	**9**	**1**
	Child abuse	**5**	**4**	**3**	**3**	**1**
**Number of past history factors by patient**		**2.1 [0–6]**	**2.1 [0–6]**	**2.3 [0–6]**	**2.0 [0–9]**	**1 [0–3]**
**Ongoing, non-psychiatric conditions**	Isolated sleeping problems	**6**	**2**	**0**	**0**	**4**
	Dementia	**2**	**1**	**0**	**0**	**1**
	Specific neuropathic pain	**3**	**1**	**1**	**1**	**2**
	Sciatic pain	**3**	**3**	**2**	**2**	**0**
	Migraine	**3**	**2**	**1**	**0**	**1**
	Fibromyalgia	**3**	**1**	**1**	**1**	**2**
	Musculo-skeletal complaints or pain	**16**	**14**	**13**	**7**	**2**
	Tension type headaches	**7**	**7**	**4**	**3**	**0**
	Isolated fatigue	**10**	**9**	**7**	**3**	**1**
	Functional gastro-intestinal complaints: Irritable bowel syndrome	**12**	**9**	**6**	**0**	**3**
	Sexual dysfunction, premature ejaculation	**5**	**4**	**3**	**3**	**1**
	Enuresis, incontinence	**3**	**3**	**3**	**2**	**0**
	Other chronic disease (e.g. Diabetes type 2)	**35**	**25**	**19**	**11**	**7**
	Chronic low back pain	**6**	**5**	**2**	**2**	**1**
**Number of non-psychiatric conditions by patient Mean/Range**		**2.28 [0–7]**	**2.09 [0–7]**	**1.75 [0–6]**	**2.0 [0–7]**	**2.77 [1-5]**
Ongoing non-psychiatric reasons	Chronic diffuse complaints	**12**	**12**	**10**	**6**	**0**
	Unexplained complaints	**13**	**11**	**8**	**3**	**2**
	Diffused pain	**8**	**7**	**4**	**2**	**1**
**All reasons and conditions by patient Mean/Range**		**4.1 [1-9]**	**4.2 [1-9]**	**3.9 [1-9]**	**4.22 [1-9]**	**3.2 [1-5]**

^1^All psychiatric conditions, including symptoms of depression without all criteria for a major episode, all diagnoses of anxiety.

^2^All depressive episodes, whatever the intensity.

^3^Major depressive episodes only.

### Objectives and outcomes

We wanted first to determine the number of AD prescriptions by the GPs, the characteristics of these prescriptions both for psychiatric and non-psychiatric conditions (name of AD, dosage and duration of treatment), and the biological and psychosocial characteristics of the patients, including the patients’ socio-professional category. Three categories - on-going problems, patient history, and biomedical conditions - were distinguished. Secondly, we assessed the influence of these determinants based on the interviews with the prescribing GPs.

Based on data regarding prescription of ADs for non-psychiatric conditions, our initial hypothesis was that a 20% prevalence of AD prescriptions not related to any psychiatric condition would be found [3,18]. The other hypothesis was that the GPs’ decision would be influenced by multiple factors related to the complexity of general practice situations.

### Analysis

Data were collected using Excel®. Any outlier and missing data were tracked. A descriptive analysis was performed using “epi-info® 3.53”. To get an indication of the weight of the influencing factors for the 50 randomly chosen patients, the sum of all individual determinant scores, rated from 0 to 2 for each patient, was calculated. The mean number and range of influencing factors per patient as well as the modus score for each influencing factor was determined. We determined the strength of influence by dividing the score of influence by the prevalence for each factor.

### Ethical aspects

The local ethics committee (CPP Nord-Ouest) stated that an authorization was not required for this non-interventional study.

## Results

### GP characteristics

Among the 56 GPs invited, 28 (6 females and 22 males) agreed to participate. The mean age was 48.5 years (32–63; SD = 9.5). Nine worked in rural practice, and 14 were mentoring students. The mean duration of practice was 17.8 years (3–35; SD = 10.5). The mean number of patients met during the week of the study was 126 (56–260; SD = 40.4).

### Overall patient characteristics

During the study week, the GPs saw 3,522 patients, 317 of whom (8.90%; [3.94 -17.02]) were prescribed an AD. Two hundred twenty-nine patients were female (72.2% [67–77.1]) and 88 (27.8% [23–33.1]) male. Their mean age was 54 [18–91]. Fifty-six per cent (n = 178) were married or living together; the others were single (n = 50 [16%]), divorced (n = 48 [15%]) or widowed (n = 41 [13%]. Most patients belonged to a low-income social class (n = 68 [52%]). Overall, 235 patients (74.1% [68.9-78.9]) were prescribed “new” ADs (SSRIs and SNRIs). For more than one in four patients, the AD treatment was initiated during the previous six months (n = 83 [26%]). The participating GPs themselves initiated 208 prescriptions (65.6 [60.1-70.8]). During the study week every GP had nearly three patients (2.96) for whom they initiated an AD prescription.

### In-depth assessment of the 50-patient sample

The results are described in Table 1. Half of the patients had no professional activity and 17 had work-related problems. Nine patients had no diagnosis of any psychiatric condition. According to their GP, the other 41 had a psychiatric diagnosis: major depressive episode, depressive episode or anxiety, or sometimes a combination. On the whole, patients had from 1 to 9 reasons or conditions, (mean = 4.1) and from 0 to 8 non-psychiatric conditions (mean = 2.94), showing a high level of multi-morbidity. Many of these conditions were related to pain.

The prevalence of the conditions assessed and their most frequent influence score are shown in Table 2. The relative influence for the most important of them is shown in Table 3. Among the contextual factors, work-related problems and recent bereavement showed both a high prevalence and strong influence on AD prescription. Within the past medical history, former AD prescription and depression were noticeably prevalent. Fibromyalgia and neuropathic pain, though not so prevalent, nonetheless had a strong influence on the decision to prescribe an AD. Among the nine patients without a psychiatric disease, the conditions often associated were fibromyalgia (n = 2), pain (n = 2), premature ejaculation (n = 1), sleeping troubles (n = 3, 2 for a withdrawal process), exhausted family caregiver (n = 1) serious grief (n = 1). Other chronic diseases for which an AD was prescribed included disability after a stroke and chronic cardiac failure.

**Table 2:** Influence, prevalence, and modus of influence of each factor (n = 50)

**Type of situation**	**Condition or characteristic**	**Total score of Influence**^**(1) **^**(n)**	**Prevalence**^**(2) **^**(n)**	**Most frequent influence score**^**(3) **^**(modus)**
**Psychiatric conditions**	Anxiety (all)	41	24	2
	Major depressive episode (intense)	40	20	2
	Depressive episode (mild)	20	11	2
	Symptoms of bad mood without depression	4	3	1
**Psychiatric-related past history**	Past history of AD prescription	30	23	2
	Past history of depression	24	19	1/2
	Family with psychiatric conditions	8	10	1
	Past history of suicide attempt	7	7	1
	Past history of excessive alcohol consumption	4	6	0
	Past history: Hospitalised in psychiatric ward	4	5	1
**Non-psychiatric conditions**	Other chronic disease (e.g.: Diabetes type 2)	22	35	0
	Musculo- skeletal complaints or pain	11	16	0
	Isolated fatigue	11	10	2
	Isolated sleeping problems	7	6	0/1
	Functional gastro-intestinal complaints: Irritable bowel syndrome	5	12	0
	Sexual dysfunction, premature ejaculation	5	5	0/2
	Specific neuropathic pain	5	3	2
	Tension type headaches	4	7	0
	Fibromyalgia	4	3	2
	Chronic low back pain	3	6	0
	Migraine	2	3	0
	Dementia	2	2	0/2
	Sciatica pain	1	3	0
	Enuresis, incontinence	0	3	0
**Non- psychiatric reasons**	Chronic complaints	13	12	1
	Unexplained complaints	10	13	1
	Diffused pain	10	8	2
**Non- psychiatric past history**	Past history of mistreatment/child abuse	5	5	1
	Past history of tobacco consumption	2	18	0
	Past history of other addictions (e.g. Gambling)	1	5	0
**Other factors**	Work-related problems	23	17	2
	Recent bereavement/Recent death in entourage	15	12	2
	Legal problems/Current legal situation	8	4	2
	Position of family care giver	7	7	1
	Physically or psychologically dependent patient	6	7	0
	Facing discrimination	6	5	1
	Social loneliness/Socially isolated patient	6	5	1
	Disability (official status)	5	8	0
	Current tobacco consumption	2	14	0
	Other addictions (Opiates, gambling, etc.)	2	6	0
	Excessive alcohol consumption	2	3	0
	Replacement therapy (methadone, buprenorphine)	0	1	0

^(1)^Total score is the sum of all individual influence scores (2 = Strong influence, 1 = possible influence, 0 = no influence) given for each condition/patient.

^(2)^Total prevalence may be > 50 as patients may have multiple conditions.

^(3)^Influence score most frequently given by the GPs. In the event of a tie, the two scores are separated by a slash (“/”).

**Table 3:** Strength of influence for the main factors

**Condition or characteristic**	Influence score (*)
Major depressive episode (intense)	2
Legal problems/Current legal situation	2
Depressive episode (mild)	1.8
Anxiety (all)	1.7
Specific neuropathic pain	1.6
Work-related problems	1.3
Symptoms of bad mood without depression	1.3
Past history of AD prescription	1.3
Past history of depression	1.3
Fibromyalgia	1.3
Recent bereavement/Recent death in entourage	1.2
Facing discrimination	1.2
Social loneliness/Socially isolated patient	1.2
Diffused pain	1.2
Isolated fatigue	1.1
Isolated sleeping problems	1.1
Sexual dysfunction, premature ejaculation	1
Past history of suicide attempt	1
Dementia	1
Past history of mistreatment/child abuse	1
Position of family care giver	1

(*) The influence was determined by dividing the score of influence by the prevalence for each factor.

## Discussion

### Prescription of ADs: characteristics

This study confirmed the high frequency of AD prescription in general practice. Nevertheless, one third of the AD prescriptions were not initiated by the GPs, but were renewals. The rate of AD prescription varied considerably, from 4% to 17% per GP, as has been observed in other studies [23]. One patient in four (26%) had recently been prescribed an AD, initiated by his own GP. During the study week, each of the GPs saw an average of 11 patients taking ADs: On average eight of the patients were already taking the AD and the physician initiated the prescription for the other three. One patient in two had had a long-term prescription, more than one year, which is consistent with other studies [10,12].

### Patient characteristics

The most prevalent and influential conditions were anxiety and depression, in contradiction with other studies [6,18]. According to their GP, 76% of the patients presented with a full psychiatric condition. Women were particularly affected. Only 6% of the patients presented isolated psychological symptoms, and 18% no psychiatric condition at all. The fact that anxiety was an important reason for AD prescription must be highlighted, though anxiety is partly associated with depression. French health authorities have stigmatised the prescription of benzodiazepines by GPs and their level of prescription has stagnated [3], which has probably produced a shift away from benzodiazepines towards more AD prescriptions. The influence of a past history of depression or previous AD prescription has also to be pointed out, as it was already suggested from our previous qualitative study [22]. Current use and past history of tobacco consumption were highly common, a phenomenon previously known among these categories of patients [24].

### The importance of psychosocial situations

In addition to already known risk factors for depression and psychiatric problems, the GPs underlined the influences of psychosocial factors. They reported that work related problems had an important influence on their decision. Legal problems, recent grief, and discrimination or social isolation also strongly influenced the GPs’ decisions. This study also confirmed previously known factors for psychological and social distress: More than half of the patients were single, divorced or widowed, and belonged to a low-income social class.

### The importance of non-psychiatric conditions

For almost 20% of the enrolled patients, the reason for an AD prescription was a non-psychiatric condition. Many of the patients suffered from numerous on-going conditions. These conditions were also present as well in the case of psychiatric diseases as a co-morbid condition. The GPs decided to prescribe ADs for chronic non-psychiatric conditions such as chronic complaints, pain, or sleep-related problems. Many of these non-psychiatric conditions (pain, migraine, fibromyalgia migraine, and premature ejaculation) met the criteria for scientific evidence for the prescription of ADs [15,25-28]. This level of evidence was lacking for some other conditions that influenced GPs, e.g. isolated sleep related problems or sciatica and low back pain, but these conditions were not the sole reason for prescription to these patients. They also had chronic ischemic artery conditions and type 2 diabetes, but the presence of these other chronic “physical” diseases was said to be of little influence. Only one patient was prescribed an AD mainly for restless legs syndrome, which is in fact a well-known side effect of ADs.

### Implications for practice

Many of these AD prescriptions, including those for non-psychiatric conditions, seemed scientifically justified. We did not find “unexplained” prescriptions, but this did not mean that all of them were fully appropriate. Thus, these results indicate areas for improvement. 

Firstly, the GPs should be aware of the influence of their patients ‘context of life, such as family or work related problems, on their prescription behaviour. A bio psycho social approach, patient’s centered, is therefore relevant in PC settings: It helps to build the relationship and to understand the patients’ perspective in cases of stressful life events. These events seem to have too much influence on the AD prescription decisions of GPs.

Secondly, at the opposite, patients with chronic diseases did not seem to trigger AD prescriptions, even though they are a well-known factor for depression, sometimes leading to higher morbidity outcomes [29]. Do GPs pay enough attention of mood disturbances amongst these patients?

Thirdly the important number of patients for whom the prescription was not initiated by the GP himself has to be underlined: We do not know how these prescriptions are re-assessed. Their appropriateness should be questioned.

### Implications for a broader study

This pilot study also specifically assessed the feasibility of a larger study. The aim will be to confirm these findings with a representative GP population sample. We sought to precisely present the rate of “prescriptions for non-psychiatric conditions”. Based on the outcome of this pilot study (i.e. an 18% rate of AD prescriptions for non-psychiatric conditions), with a 95% confidence interval, a sample of 600 AD prescriptions will be required in order to obtain a minimum of 120 non-psychiatric prescriptions. With three prescriptions assessed for each GP, an intra-cluster correlation coefficient of 0.01 must be taken into account. The required sample of patient files and GPs will be 612 and 204 respectively. As a result, the mean number of patients eligible per week for the main study can roughly be estimated at three per GP per week. Taking into account Lasagna’s law for prevalence studies, we can assume that two eligible patients per week is probably more realistic [30].

### Implication for further research

We observed that some GPs prescribed ADs four times less frequently than others. Many hypotheses can be made. Differences in consumption could be related to differences between areas or countries [5]. Urbanization could influence the prevalence of mood disorders [31]*.* Patient characteristics, physician characteristics, the health care system, and interactions between these three components, all influence AD prescription [32]. The medical curriculum can influence the ability of the GPs to recognize and treat mood disorders [33]*.* In a full study, the influence of the GPs’ practice location, their conviction regarding the efficacy of ADs, the number of patients seen, and the organization of the practice (solo or group practice) on AD prescription rate should be explored. Two other problems should be taken into account, i.e. a possible memory bias and under-recording of AD prescriptions. To ensure representativeness, the rate of AD prescriptions during four weeks of inclusion will be compared with the rate of annual AD prescription according to the French social security system. One challenge will be to understand differences in the rates of AD prescription by individual GPs and to interpret their prescription behaviour. This study, using a mixed-method design, will provide the chance to set up an opportunistic sample to perform qualitative interviews among these GPs in order to disclose their conception of care and their style of practice that potentially influences their AD prescriptions.

### Strengths and weaknesses

This study assessed the influence of all patient characteristics according to the GPs, but was not designed to assess the reliability of their diagnosis with a standardized questionnaire. Thus, the level of appropriateness of AD prescription for depression and anxiety should be interpreted with caution. Psychiatric co morbidities are frequently associated in depressed people. Personality disorders could range for 25% of the patients treated with ADs. The presence of these disorders seems to negatively impact treatment outcomes [34]. In our study, the GPs disclosed prescription for several patients having “symptoms of depression” without all the items of the DSM 5 major depressive episode. Only a precise assessment of the patient‘s symptoms and personality could have provided an answer on fully appropriate or inappropriate AD prescriptions. Nevertheless this is a difficult challenge and subject of debate among psychiatrists. Medicalization of sadness in the context of our society is also an important issue, as people complain but are not being assessed as depressed by standardized instruments. Horwitz pointed out the changing conception of sadness and depression in our modern world [35]. For him, the limits of the concept of “depressive disorder” are vague. In the context of medicalization of life problems, it is tempting for the GP to analyse complaints only from a medical point of view. This could lead them to treat only symptoms of anxiety or sadness as a mental disorder.

Another possible bias could be different ways of practice among the GP participants: It is likely that GPs mentoring students are more prone to follow EBM guidelines. Such a behaviour could lower the rate of overall AD prescription and change the relative weight of the factors influencing the AD prescription. Nevertheless, the figures on the rate of prescription for non-psychiatric conditions were consistent with the range of calculations made based on the available literature. Collaboration of the GPs, availability of the data, and comprehensibility of the questionnaire were ensured, which is consistent with good internal and external validities. This is a pilot study: All of these data have allowed us to precisely design the protocol of a full study that will be done in order to validate these preliminary results in a larger population.

## Conclusion

The mean rate of AD prescription by GPs was nearly 9%. Among these, nearly 20% were prescriptions for non-psychiatric conditions. Non-psychiatric prescriptions were mainly related to complex contextual situations and multi-morbid patients. These preliminary results emphasise the influence of social and psychological factors in the GP’s decision for this prescription. This pilot study ensures the feasibility of our upcoming study that aims to more precisely assess the reasons for and influences on this prescription behaviour.

## Competing interests

The authors declare they have no conflict of interest.

## Authors’ contributions

Conception of the idea for the study: AM. Development of the protocol: AM, IAA, JPL, MS, PB. Data collection and extraction: AM, IAA, JPL. Critical assessment: AM, IAA, JPL, PB. PVR and LP participated in the design and coordination and helped to draft the manuscript. Writing of the manuscript: AM. All of the authors have read the draft critically, to make contributions, and have approved the final text.
